# A scoring system predicting acute radiation dermatitis in patients with head and neck cancer treated with intensity-modulated radiotherapy

**DOI:** 10.1186/s13014-019-1215-2

**Published:** 2019-01-21

**Authors:** Mitsue Kawamura, Michio Yoshimura, Hiromi Asada, Mitsuhiro Nakamura, Yukinori Matsuo, Takashi Mizowaki

**Affiliations:** 1 Department of Radiation Oncology and Image-applied Therapy, Graduate School of Medicine, Kyoto University, 54 Kawahara-cho, Shogoin, Sakyo-ku, Kyoto, Kyoto 606-8507 Japan; 2 Nursing Department, Kyoto University Hospital, Kyoto, Japan; 3 Division of Medical Physics, Department of Information Technology and Medical Engineering, Human Health Sciences, Graduate School of Medicine, Kyoto University, Kyoto, Japan

**Keywords:** Head-and- neck cancer, IMRT, VMAT, Skin dose

## Abstract

**Background and purpose:**

We created a scoring system incorporating dosimetric and clinical factors to assess the risk of severe, acute skin reactions in patients undergoing intensity-modulated radiation therapy (IMRT) to treat head and neck cancer (HNC).

**Materials and methods:**

A total of 101 consecutive patients who received definitive IMRT or volumetric modulated arc therapy (VMAT) with a prescription dose of 70 Gy to treat HNC between 2013 and 2017 in our hospital were enrolled. Skin V_5Gy_, V_10Gy_, V_20Gy_, V_30Gy,_ V_40Gy_, V_50Gy_, and V_60Gy_ values delivered 5 mm within the body contour were compared between patients with Grades 1–2 and Grade 3 dermatitis. A scoring system was created based on logistic regression analysis (LRA) that identified the most significant dosimetric and clinical factors.

**Results:**

The V_60Gy_ was significantly associated with radiation dermatitis grade in both LRA and recursive partitioning analysis (RPA). A scoring system incorporating the V_60Gy_, concurrent chemotherapy status, age, and body mass index was used to divide all patients into three subgroups (0–1, 2–3, and 4–6 points) in the RPA. The incidence of Grade 3 dermatitis significantly differed among the subgroups (0, 20.5, and 58.6%, respectively, *P* < 0.01).

**Conclusions:**

A risk analysis model incorporating dose-volume parameters successfully predicted acute skin reactions and will aid in the appropriate management of radiation dermatitis.

## Highlights

A scoring system predicting radiation dermatitis.

## Introduction

Radiotherapy is a principal treatment for head and neck cancer (HNC). Definitive radiotherapy in patients with advanced HNC can preserve laryngopharyngeal functions such as speaking, swallowing, and breathing. Radiotherapy for HNCs must minimise exposure to a large number of organs-at-risk (OARs). Intensity-modulated radiotherapy (IMRT) reduces complications and escalates dose delivery [1]. The radiation dose necessary to achieve local tumour control is limited by doses to normal tissues within the irradiation field [2, 3]. Radiation dermatitis is one of the most common adverse events associated with head and neck (HN) radiotherapy, and is radiation dose-dependent [4]. During two- or three-dimensional conformal radiotherapy (2D/3D-CRT), dose distribution to the skin is homogeneous and can be easily estimated, but the radiation dose delivered by IMRT is inhomogeneous and difficult to calculate. The significance of dose-volume factors when performing radiotherapy is well recognised, and it is preferable to minimise the skin dose, but no clear indication of the severity of acute skin reactions is available. Radiation dermatitis has a profound impact not only on quality of life (QOL) but also on treatment outcomes; radiation schedules may be interrupted [5–7]. Thus, prediction of radiation dermatitis risk is essential for appropriate management. A previous report suggested several predictors of acute toxicities [8]; our current study includes both dose-volume parameters and other clinical factors.

Here, we evaluated the relationship between skin dose distributions and the incidence of severe, acute radiation dermatitis in HNC patients treated with IMRT and volumetrically modulated arc therapy (VMAT). We developed a scoring system combining dose parameters with clinical factors that usefully predicted acute skin reactions, facilitating the appropriate management of radiation dermatitis.

## Materials and methods

### Patient population

We retrospectively identified 101 consecutive patients with HNC, all of whom underwent definitive radiotherapy of 70 Gy between 2013 and 2017 in our hospital. Patient characteristics are shown in Table 1. Classification by tumour histology revealed 99 squamous cell carcinomas, 1 adenocarcinoma, and 1 myoepithelial cancer. Two patients were of stage I, 12 were of stage II, 18 were of stage III, and 69 were of stage IV; staging was done using the malignant tumour criteria of the Union for International Cancer Control (7th edition) [9]. IMRT using a 4 MV photon beam was used to treat 46 patients from 2013 to 2014, and VMAT employing a 6 MV photon beam was used to treat 55 patients from 2014 to 2017. The median overall treatment time was 50 days (range, 46–62 days). Written informed consent was obtained from all patients, and the study was approved by our local ethics committee.

**Table 1:** Patient characteristics

Age (years)	24–84	(median 67)	67)		
Sex					
Male	75				
Female	26				
Tumor site					
Tongue	5				
Gingiva	7				
Nasopharynx	14				
Oropharynx	34				
Hypopharynx	24				
Larynx	6				
Nasal cavity	1				
Paranasal sinuses	9				
Unknown	1				
Histology					
Squamous cell carcinoma	99				
Other	2				
TNM stage		N0	N1	N2	N3
	T0–1	2	2	8	3
	T2	9	3	23	3
	T3	10	3	6	1
	T4	5	5	18	0
Treatment					
Neoadjuvant (+)	41				
Neoadjuvant (−)	60				
Concurrent (+)	78				
Concurrent (−)	23				
Radiation treatment					
IMRT-4X	46				
VMAT-6X	55				

Abbreviations: *IMRT* intensity modulated radiotherapy, *VMAT* volumetric modulated arc therapy

Neoadjuvant therapy: TPF (Docetaxel, Cisplatin, 5-Fluorouracil), TPE (Docetaxel, Cisplatin, Cetuximab), FP (Cisplatin, 5-Fluorouracil), CDDP (Cisplatin), and CBDCA (Carboplatin)

Adjuvant therapy: CDDP (Cisplatin), CBDCA (Carboplatin), and Cet (Cetuximab)

### IMRT and VMAT

All patients were immobilised in the supine position with fixation mask and scanned over the neck and upper thorax using the LightSpeed RT Computed Tomography (CT) platform (2.5 mm thick slices; GE Healthcare, Madison, WI, USA). The critical structures and target volumes were delineated by radiation oncologists and medical physicists on axial slices. In line with ICRU Reports 50 and 62 [10, 11], the gross tumour volume (GTV) was defined as the gross extent of tumour evident in CT images, including both the primary tumour and gross regional LNs. The clinical target volume (CTV) was defined as the GTV plus a margin allowing for potential microscopic tumour extension and encompassing the adjacent regional LNs. The planning target volume (PTV) was the CTV plus a 5 mm wide margin to allow for uncertainties in radiation delivery, the internal margin, and the set-up margin. The PTV70Gy volume included the primary tumour and LN metastases, the PTV63Gy included the high-risk LNs, and the PTV56Gy included the low-risk LNs. All PTVs were clipped from the body contours by 3 mm to reduce the skin doses [12]. The principal OARs were the spinal cord, brainstem, both parotid glands, and the oral cavity. Critical organs (the brainstem and the spinal cord) were assigned 5 mm margins when generating planning risk volumes. The nominal energies of the flattened photon beams of Clinac 6EX and Clinac iX (Varian Medical Systems, Washington DC, USA) were 4 and 6 MV, respectively. Dose calculations were performed using Acuros XB software (ver. 13.7.14; Varian). The simultaneous integrated boost technique was used to deliver 70, 63, and 56 Gy in 35 fractions to the PTV70Gy, PTV63Gy, and PTV56Gy, respectively. The dose constraints of targets, and the OARs used to optimise the IMRT and VMAT plans, met our institutional criteria (Table 2).

**Table 2:** Planning constraints

Structure	Index	Objectives	Acceptable
PTV70Gy	D50% (%)	100%	98–103%
PTV70Gy	D98% (%)	> 93%	> 90%
PTV70Gy	D2% (%)	< 105%	< 115%
PTV63Gy	D90% (Gy)	100% (63 Gy)	> 97% (61.11Gy)
PTV63Gy	D50% (Gy)	< 105% (66.15 Gy)	< 108% (68.04Gy)
PTV56Gy	D90% (Gy)	100% (56 Gy)	> 97% (54.32Gy)
PTV56Gy	D50% (Gy)	< 105% (58.8 Gy)	< 108% (60.48Gy)
CTV70Gy	D95% (%)	> 100%	> 98%
CTV63Gy	D95% (Gy)	> 100% (63 Gy)	> 98% (61.74Gy)
CTV56Gy	D95% (Gy)	> 100% (56 Gy)	> 98% (54.88Gy)
GTV	D95% (%)	> 100%	> 98%
Spinal cord	Max	45 Gy	50 Gy
Brain stem	Max	54 Gy	60 Gy
Contralateral parotid grand	V30Gy	< 50%	< 50%
Oral cavity	Mean	30 Gy	< 40 Gy

Abbreviations: *DXX%* dose to xx% of the organ; *V30Gy* volume receiving 30 Gy

### Skin evaluation

Both board certificated radiation oncologists and otolaryngologists scored all incidents of acute radiation dermatitis weekly during treatment and 1 month after treatment using the Common Terminology Criteria for Adverse Events (CTCAE) ver. 4.0 [13]. The highest grade of toxicity served as the reference value. The CTCAE for dermatitis defined Grade 1 as faint erythema or dry desquamation; Grade 2 as moderate to brisk erythema, patchy moist desquamation (mostly confined to skin folds and creases), and moderate edema; Grade 3 as moist desquamation in areas other than skin folds and creases, and bleeding induced by minor trauma or abrasion; and Grade 4 as life-threatening skin necrosis or ulceration of the full-thickness dermis, spontaneous bleeding, and a need for skin grafts.

### Dose-volume histogram analyses

An irradiation boundary of 5 mm inside the body contour was automatically generated at a threshold of − 350 HU to evaluate skin structure using the definition of the Radiation Therapy Oncology Group. Then dose-volume histograms (DVHs) were calculated for the skin, which served as a surrogate for the epidermis and dermis.

### Statistical analyses

Statistical analyses were performed using EZR ver. 1.31 software (Saitama Medical Center, Jichi Medical University, Saitama, Japan), which is a graphical user interface for R (the R Foundation for Statistical Computing, Vienna, Austria) [14]. The relationship between DVH parameters and the acute effects on normal tissue were compared using the Mann–Whitney U-test. The DVHs yielded the absolute volumes of V_5Gy_, V_10Gy_, V_20Gy_, V_30Gy_, V_40Gy_, V_50Gy_, and V_60Gy_. V_*d* Gy_ is the absolute volume of skin that received more than the threshold dose of *d* Gy. To evaluate the acute effects, patients were subdivided by their CTCAE scores. The effects of chemotherapy, treatment technique, sex, age, and body mass index (BMI) were also recorded. The optimal cut-off used to divide patients into two subgroups based on the radiation dermatitis grade was defined using recursive partitioning analysis (RPA). For dose-volume parameters, threshold cut-offs were used to divide the population. We also evaluated chemotherapy status, treatment technique, sex, age, and BMI via logistic regression analysis (LRA) within multivariate technique. We used the median age and lean BMI (< 18.5 kg/m^2^) to divide the population. Patients were scored by reference to the V_60Gy_, concurrent chemotherapy status, age, and BMI, all of which were significant in LRA. Radiation dermatitis scores were also recorded. After dividing the groups via RPA, the rates of Grade 3 dermatitis were compared using the Fisher’s exact test. *P* values less than 0.05 were considered statistically significant.

## Results

**Fig. 1:**
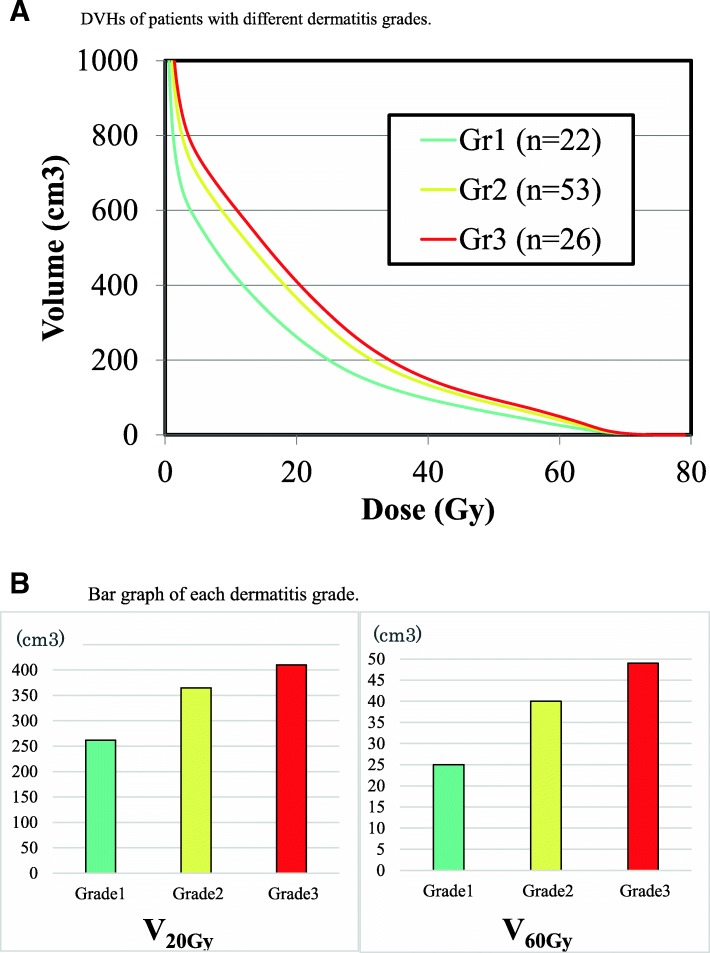
**a** DVHs of patients with different dermatitis grades. **b** Bar graph of each dermatitis grade

Average DVHs were drawn for each patient and compared to the acute skin dermatitis grade, which was our clinical endpoint (Fig. 1a). Grade 1 dermatitis was observed in 22 patients, Grade 2 was observed in 53, and Grade 3 was observed in 26; no patient had Grade 4 or 5 disease. The average skin V_20Gy_ and V_60Gy_ values were 354.4 (range, 50.3–546.0), and 39.0 (range, 2.9–88.7 cm^3^). The means, standard deviations, and *P*-values for all parameters of each grade are shown in Table 3.

**Table 3:** Comparisons of skin dose parameters among patients with Grades 1–3 dermatitis

Skin (mean ± SD)	Grade 1	Grade 2	Grade 3	*P*-value
V_5 Gy_ (cm^3^)	566 ± 224	693 ± 136	745 ± 85	< 0.01
V_10 Gy_ (cm^3^)	438 ± 197	568 ± 119	620 ± 81	< 0.01
V_20 Gy_ (cm^3^)	262 ± 128	365 ± 92	410 ± 73	< 0.01
V_30 Gy_ (cm^3^)	152 ± 75	216 ± 68	247 ± 48	< 0.01
V_40 Gy_ (cm^3^)	96 ± 45	133 ± 47	149 ± 28	< 0.01
V_50 Gy_ (cm^3^)	59 ± 30	83 ± 33	95 ± 24	< 0.01
V_60 Gy_ (cm^3^)	25 ± 17	40 ± 22	49 ± 16	< 0.01

Abbreviations: *SD* standard deviation, *V*_*dGy*_ volume of the skin that received more than the threshold dose of d Gy

*P*-value: derived by Mann–Whitney U-test comparisons between Grades 1–2 and Grade 3 patients

### Association between skin DVHs and acute dermatitis

The volumes of DVHs at each dose were related to the incidence of Grade 3 dermatitis. The Mann–Whitney U-test indicated a significant difference between Grade 1–2 and 3 patients. Comparison of the DVHs showed that patients who developed worse skin reactions received higher radiation skin dose, and that the skin dose was associated with a greater risk of acute dermatitis. Although the DVHs tended to differ at low doses of 10–20 Gy (Fig. 1a), the percentage differences were greater in the high-dose area (60 Gy) as revealed by the bar graph (Fig. 1b). The optimal cut-offs used to divide the patient population into two subgroups based on radiation dermatitis grade are shown in Table 4. The RPA and LRA showed that all dose parameters were significant, particularly the V_60Gy_. The probability of developing dermatitis in each grade by chemotherapy status, treatment technique, sex, age, and BMI are shown in Table 5.

**Table 4:** Optimal cut-off values for and crude rates of Grade 3 radiation dermatitis

	Cut-off value	Grade 3 radiation dermatitis < Cut-off > Cut-off		*P-*value
V_5Gy_ (cm^3^)	690	5/40 (12.5%)	21/61 (34.4%)	0.019
V_10Gy_ (cm^3^)	565	5/40 (12.5%)	21/61 (34.4%)	0.019
V_20Gy_ (cm^3^)	400	9/66 (13.6%)	17/35 (48.6%)	< 0.01
V_30Gy_ (cm^3^)	190	2/38 (5.3%)	24/63 (38.1%)	< 0.01
V_40Gy_ (cm^3^)	115	2/34 (5.9%)	24/67 (35.8%)	< 0.01
V_50Gy_ (cm^3^)	85	8/54 (14.8%)	18/47 (38.3%)	0.011
V_60Gy_ (cm^3^)	38	6/55 (10.9%)	20/46 (43.4%)	< 0.01 ^a^

Abbreviations: *V*_*xxGy*_ volume of the skin that received xx Gy

^a^V_60Gy_ was the most significant dose parameter in both LRA and RPA

**Table 5:** Effects of non-dose parameters on dermatitis grade

	Grade 1 (*n* = 22)	Grade 2 (*n* = 53)	Grade 3 (*n* = 26)	Total
Neoadjuvant chemotherapy				
(−)	17 (28.3%)	34 (56.7%)	9 (15.0%)	60
(+)	5 (12.2%)	19 (46.3%)	17 (41.5%)	41
Concurrent chemotherapy				
Radiotherapy alone	6 (26.1%)	15 (65.2%)	2 (8.7%)	23
Platinum	12 (22.6%)	29 (54.7%)	12 (22.6%)	53
Cetuximab	4 (16.0%)	9 (36.0%)	12 (48.0%)	25
Treatment technique				
IMRT-4X	7 (15.2%)	23 (50.0%)	16 (34.8%)	46
VMAT-6X	15 (27.3%)	30 (54.5%)	10 (18.2%)	55
Sex				
Male	16 (20.8%)	37 (51.4%)	22 (27.8%)	75
Female	6 (24.1%)	16 (58.6%)	4 (17.2%)	26
Age (years)				
< 67	10 (18.2%)	32 (58.2%)	13 (23.6%)	55
**≥** 67	12 (26.1%)	21 (45.7%)	13 (28.3%)	46
BMI (kg/m^2^)				
< 18.5	2 (8.0%)	14 (56.0%)	9 (36.0%)	25
**≥** 18.5	20 (26.3%)	39 (51.3%)	17 (22.4%)	76
Diabetes				
Yes	3 (23.0%)	7 (53.9%)	3 (23.1%)	13
No	19 (22.0%)	46 (52.3%)	23 (26.1%)	88
Hypertension				
Yes	7 (23.0%)	18 (60.0%)	5 (16.7%)	30
No	15 (21.0%)	35 (49.3%)	21 (29.6%)	71
Any comorbidity				
(Charlson Comorbidity Index> 1)				
Yes	1 (10.0%)	5 (50.0%)	4 (40.0%)	10
No	21 (23.0%)	48 (52.8%)	22 (24.2%)	91
Smoking				
Concurrent	7 (17.0%)	20 (48.8%)	14 (34.1%)	41
Ex	7 (19.0%)	21 (56.8%)	9 (24.3%)	37
Never	8 (35.0%)	12 (52.2%)	3 (13.0%)	23

Abbreviations: BMI = body mass index

### Scoring system for, and risk classification of radiation dermatitis

**Fig. 2:**
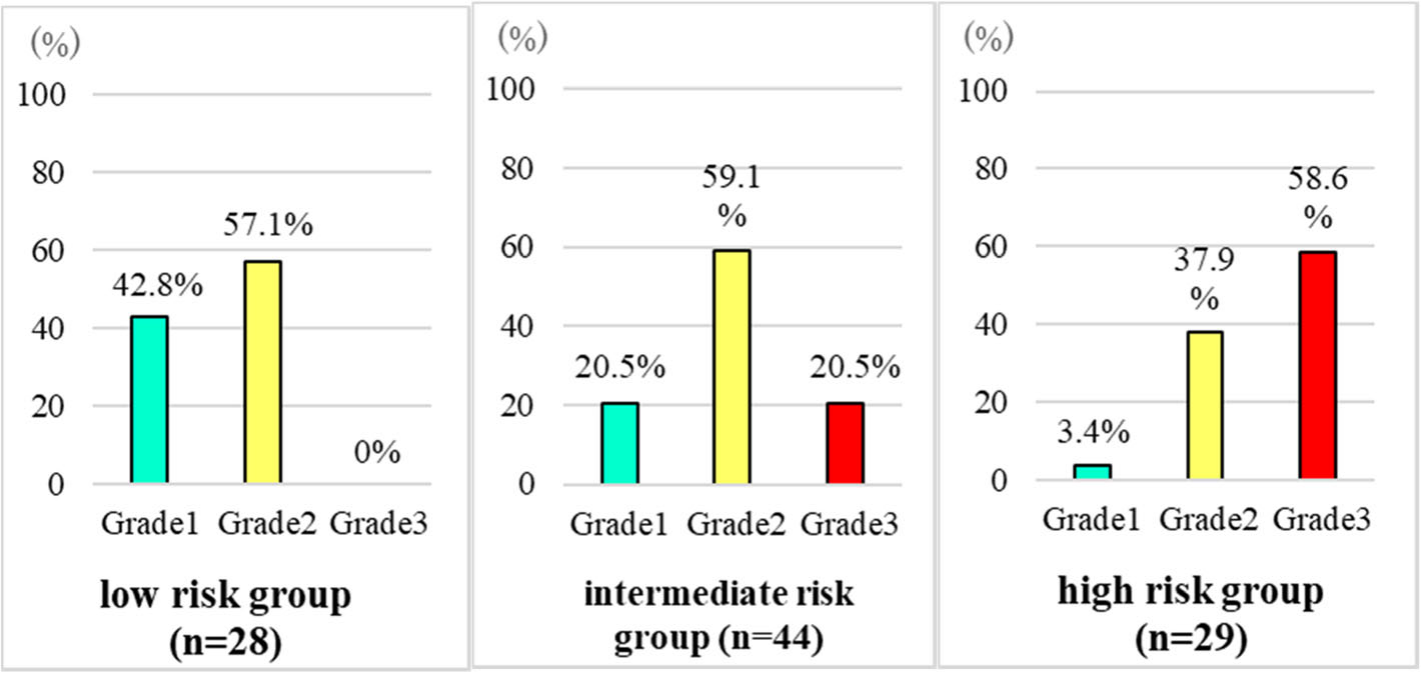
RPA by risk classification score

**Fig. 3:**
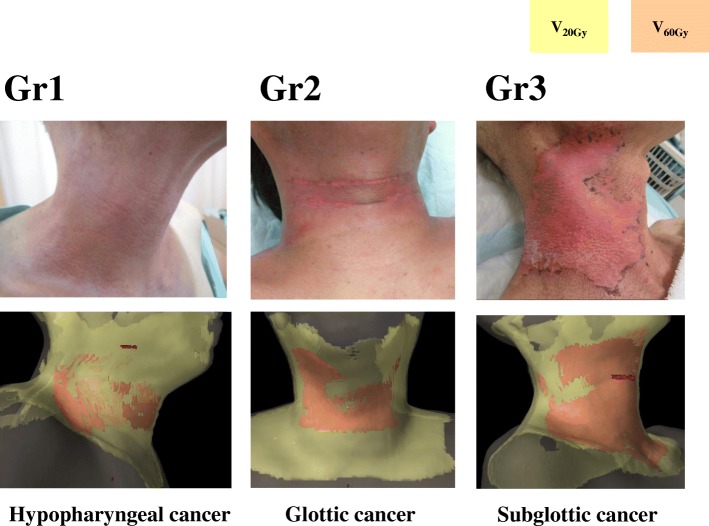
Representative images of patients with dermatitis of various grades. Skin that received 20 Gy (V_20Gy_): yellow; V_60Gy_: orange

All of the V_60Gy_, concurrent chemotherapy, age, and BMI were significant in LRA. The estimated values of V_60Gy_, BMI, age, and platinum and cetuximab chemotherapy were 1.8311, 0.9547, 0.8699, 1.1227, and 2.2713, respectively (Table 6). LRA showed that a V_60Gy_ > 40.3 cm^3^ scored 2 points, BMI < 18.5 kg/m^2^ scored 1 point, age ≥ 67 years scored 1 point, platinum therapy scored 1 point, and cetuximab therapy scored 2 points (Table 7). Using this scoring system, patients were divided into three subgroups (0–1 points, 2–3 points, and 4–6 points) in the RPA; the incidences of Grade 3 dermatitis of low, intermediate, and high risk group were 0, 20.5, and 58.6%, while those of Grade 1 dermatitis were 42.8, 20.5, and 3.4%, respectively (*P* < 0.01) (Table 6, Fig. 2). Representative images of patients with each grade are shown in Fig. 3.

**Table 6:** Factor estimates as determined by LRA

	Estimate	*P*-value	Score assigned
V_60Gy_ (≥ 38 cm^3^)	1.8311	< 0.01	2.0
BMI (< 18.5 kg/m^2^)	0.9547	< 0.01	1.0
Age (≥67 years)	0.8699	< 0.01	1.0
Concurrent chemotherapy			
Platinum	1.1227	< 0.01	1.0
Cetuximab	2.2713	< 0.01	2.0

**Table 7:** Radiation dermatitis scoring system

	0 point	1 point	2 points
V_60Gy_ (cm^3^)	< 38		≥ 38
BMI (kg/m^2^)	≥18.5	< 18.5	
Age (years)	< 67	≥67	
Concurrent chemotherapy	None	Platinum	Cetuximab

## Discussion

We evaluated the relationship between skin dose-volume distributions and the incidence of severe, acute radiation dermatitis in HNC patients treated with IMRT and VMAT. We constructed a risk analysis model combining dose parameters with concurrent chemotherapy, age, and BMI. The model usefully predicted acute skin reactions, facilitating the appropriate management of radiation dermatitis.

Acute dermatitis is the most common side effect of HNC radiotherapy, and usually develops within 90 days of exposure [15]. The skin of the anterior neck is the most sensitive region of the body [16]. During radiotherapy, ionisation of cellular water and generation of short-lived free radicals trigger irreversible double-stranded breaks in nuclear and mitochondrial DNA, as well as inflammation [17–19]. Repeated exposure to low-dose ionizing radiation prevents DNA and tissue repair. Accumulation of radiation-induced changes in the dermal vasculature, appendageal structures, and epidermal stem cells results in dermatitis progression through characteristic stages that increase in severity. Radiation dermatitis is a dose-dependent toxic effect. The total dose, dose per fraction, and dose volume to surfaces exposed to radiation affect radiation dermatitis risk. In the era of low-energy techniques, skin changes depend on the radiation dose (erythema after doses ≥2 Gy; dry desquamation after doses of 12–20 Gy, moist desquamation at doses > 20 Gy, and necrosis at doses ≥35 Gy). In terms of necrosis/ulceration endpoints, the skin TD5/5 (the 5% probability of a complication within 5 years of treatment) was 55 Gy for a 100 cm^2^ field. The TD3/5 was 57 Gy for a 30 cm^2^ field and 69 Gy for a 10 cm^2^ field [20]. These data revealed dose dependency of late skin reaction, but they cannot be extrapolated to acute dermatitis. It was needed to build the new indication of acute dermatitis with dose volume analysis. In our study V_60Gy_ over 38cm^3^ related to 43.4% of Grade 3 dermatitis, and it could be a new indication to predict the severity of radiation dermatitis induced by head and neck IMRT/VMAT.

IMRT delivers radiation to the planned treatment volume while minimizing the dose to normal tissues outside the target, thus reducing skin reactions. Using breast IMRT, a multicenter randomised trial showed that fewer patients in the IMRT than the conventional radiotherapy group experienced moist desquamation (31% vs. 48%) [21]. In HNC patients receiving IMRT, skin dose accumulation markedly increased (reflecting the intrinsic dose distribution profile) if no correction was applied in terms of inverse planning optimisation after initial clinical implementation [12]. When a thermoplastic mask was used for immobilisation, the skin doses were higher than those delivered without a mask because of a bolus effect. By contouring the skin as an OAR during optimisation, the volumes of neck skin receiving > 45 Gy and > 55 Gy fell to 58 and 17% of the initial values, respectively. Penoncello et al. [22] reported that VMAT is associated with fewer skin reactions than IMRT and is better at reducing skin doses. In addition, integrated boost regimens trigger higher skin doses, particularly to the shoulders, compared to traditional boost regimens. Price et al. found that a 4 mm PTV-to-skin distance minimised the odds ratio for developing superficial “hot spots” to < 1.1 when high-conformal rotational techniques such as VMAT were applied [23]. It has been suggested that the PTVs should be cropped 3 mm below the contoured body surface to prevent optimisation issues in build-up regions, except when the skin is part of the CTV (ICRU Report 83; [24]). Treatment protocol 1015 of the Japan Clinical Oncology Group, derived from a clinical trial of concurrent chemoradiotherapy to treat nasopharyngeal cancer, suggests cropping 2–3 mm below the body surface [25]. During real-life IMRT, we do not constrain the skin contour or draw DVHs, but potential skin issues should be discussed with high-risk patients. Many anti-cancer agents enhance the sensitivity to radiotherapy and may increase cellular damage and hinder tissue repair. Conventional chemotherapeutic agents and anticancer therapies featuring EGFR inhibitors increase the risk of severe radiation dermatitis. Giro et al. reported that a high rate of severe dermatitis was observed during radiotherapy combined with concurrent chemotherapy, and recommended treatment interruption if confluent moist desquamation develops at a total dose < 40 Gy [26]. Radiation dermatitis profoundly impacts the QOL, causing pain, infections, and bleeding, and also compromises treatment outcomes because of interruption to radiation schedules [4–6]. Appropriate management by risk classification is essential.

Generally, patients with Grade 1 radiation dermatitis are treated nonspecifically via general prevention measures. Dry desquamation can be treated with hydrophilic moisturisers, and pruritus and irritation respond to low- to mid-potency steroids. For patients with Grade 2–3 dermatitis featuring moist desquamation, treatment should be directed towards prevention of secondary infection and dressing the desquamation [27–30]. Treatment of each dermatitis grade requires a multidisciplinary approach, involving a radiation oncologist, nurse, wound specialist, and dermatologist.

In our study, BMI, age, and concurrent chemotherapy were significant predictor as other factors of acute toxicities. Treatment technique, sex, co-existing morbidities, diabetes, hypertension, smoking were not significant. Low Nutritional status and older age may delay wound repair, and concurrent chemotherapy enhances radiosensitizing effect, which increase not only therapeutic effects but also adverse events. A previous study reported the predictors of acute radiotherapeutic toxicities include sex, performance status, and nutritional status, and TNM stage [8]. Sex and TNM stage reflect RT doses in this previous study, so dose parameter and nutritional status are compatible with our study.

To the best of our knowledge, this is the first DVH-based analysis of how skin structure combined with other factors predicts the risk for acute dermatitis in HNC patients undergoing IMRT. During 2D/3D-CRT, the skin dose is homogeneous and can be estimated, but the dose is not homogeneous during IMRT, and conversion of isodoses to structure-level doses is required. No clear dose indications in terms of risk for severe, acute skin reactions are available. Our model calculates the probability of severe acute dermatitis for individual patients. Of all patients, 58.6% of high-risk, 20.5% of intermediate-risk, and 0% of low-risk patients developed severe acute dermatitis. Using dose parameters, and clinical factors including BMI, age, and concurrent chemotherapy status, we predicted the development of acute skin reactions more accurately than that predicted using dose parameters alone, facilitating the appropriate management of radiation dermatitis.

This scoring system should be evaluated prospectively or validated on retrospective multicentre cohort prior to implementation into clinical practice. In addition, our model should be expanded to include other physical parameters or genotypic data to improve sensitivity and specificity. Finally, an objective dermatitis evaluation method is needed, as CTCAE is subjective. One potential option is real-time laser Doppler flowmetry, which quantitatively detects changes in cutaneous microcirculation reflecting radiation-induced skin injury; use of this method should be investigated in the future [30].

## Conclusions

We generated a new risk analysis model including dose-volume and other parameters, which successfully predicted the development of acute skin reactions. Thereby this model could be useful to facilitate the appropriate management of radiation dermatitis induced by head and neck IMRT/VMAT.
